# “What’s in a name?”: Exploring inconsistent and contradictory definitions and clinical guidelines for hypertensive disorders of pregnancy from published literature from Nigeria and Bangladesh

**DOI:** 10.7189/jogh.10.010306

**Published:** 2020-06

**Authors:** Karen Kirk, Charlotte Warren

**Affiliations:** 1 Population Council, New York, New York, USA; 2 Population Council, Washington, D.C., USA

Pre-eclampsia and eclampsia (PE/E), among other hypertensive disorders of pregnancy (HDPs), are the second most common causes of maternal death globally, leading to 76 000 maternal deaths yearly [1]. These conditions are preventable and manageable, but only if they can be detected, and diagnosed early. In Nigeria, 29 percent of maternal deaths in tertiary facilities are caused by HDPs, including PE/E [2], and in Bangladesh 20 percent of maternal deaths are caused by eclampsia.[3] The World Health Organization defines PE as new onset of “hypertension during pregnancy (with persistent diastolic blood pressure (dBP) >90 millimeters of mercury, mmHg) with the occurrence of substantial proteinuria (>0.3 g /24hr)” and eclampsia as “generalized seizures in women with pre-eclampsia” [4].

In three-phase systematic literature reviews of peer-reviewed publications that were part of a landscape analysis exploring the gaps in prevention, detection, and management of PE/E in Nigeria and Bangladesh, researchers observed no consistency among terms describing HDPs in addition to the clinical definitions for diagnosis. Considering these examples from two diverse countries, we see that imprecise, contradictory definitions and clinical diagnosis criteria for HDPs present a major challenge for the global health community. Agreeing on simple definitions and clear clinical protocols is paramount; while population-specific considerations – including possible biological differences – remain challenges.

## A LOOK AT THE LITERATURE

Published peer-reviewed articles from the previous fifteen years on HDPs in Bangladesh and Nigeria reveal a multitude of terms describing and defining HDPs, with seventeen terms offering explicit definitions and clinical diagnosis criteria (**Table 1** and Table S1 in the **Online Supplementary Document**).

**Table 1:** Comparison of diagnostic requirements for pre-eclampsia, from published literature in Bangladesh and Nigeria*

	Nigeria										Bangladesh														
	Adeyinka et al., 2010	Akiibinu et al., 2013	Arinola et al., 2006	Chigbu et al., 2009	Glew et al., 2005	Glew et al., 2004	Ikechukwu et al., 2012	Ikpen et al., 2012	Salako et al., 2003	Vanderjagt et al, 2004	Akhtar et al., 2013	Begum & Fardousi, 2014	Begum et al., 2013	Bhowmik, 2013	Fatima et al, 2013	Fronczak et al., 2005	Hoque et al., 2008	Jesmin et al., 2011	Khan et al., 2014	Khatun et al., 2000	Kishwara et al., 2011	Rahman et al., 2012	Razzaque et al., 2005	Sarwar et al., 2013	Yousuf et al., 2011
																									
**Blood pressure:**																									
sBP≥140	●			●	●	●	●	●	●	●	●	●		●			●	●	●	●				●	●
sBP≥130			●						●																
sBP = 169 ± 26.0 mm Hg		●																							
dBP 90	●		●	●	●	●	●	●		●	●	●		●			●	●	●	●				●	●
dBP = 102 ± 11.0 mm Hg		●																							
or relative increase in sBP≥30 mm Hg				●					●	●								●		●					
or relative increase in dBP>15 mm Hg				●					●	●								●		●					
dBP≥110 at any time OR mean arterial pressure >105 mm Hg				●																					
“Hypertension”													●			●						●			
“Elevated blood pressure”															●						●		●		
**Timing of BP measurement:**																									
Not described		●			●	●				●		●	●	●	●	●			●		●	●	●		●
Two times	●		●	●			●	●	●								●	●		●				●	
4 h apart	●							●																	
6 h apart			●	●					●								●	●		●				●	
**Proteinuria:**																									
300 mg (0.3 g)	●													●			●	●	●	●					
2+ Dipstick								○																	
>100 mg/d			●																						
368 ± 39 mg/24h		●																							
≥190 mg/g creatinine					●	●																			
≥190 mg/g total protein										●															
or 1+ if specific gravity was ≤1.030 and pH was <8								○																	
“Proteinuria”				●			●				●	○	○		●						●	●	●	●	●
Not specified									●																
**Gestational age:**																									
After 20 weeks		●		●				●							●		●	●	●		●	●		●	●
After 22 weeks							●																		
Second half of pregnancy	●																								
Third trimester																●									
Not specified			●		●	●			●	●	●	●	●	●						●			●		
**Other maternal conditions:**																									
Epigastric/abdominal pain		●				●																			
Severe headache		●														○									
Dizziness																○									
Cerebral or visual disturbances						●																			
Vomiting		●																							
Cyanosis (bluish skin discoloration)						●																			
Pulmonary edema						●																			
Swelling/edema										●	○					○			○				○	○	
Organ dysfunction												○													
Reduction in plasma volume																					●				
Increase in peripheral resistance																					●				
Generalized vasoconstriction																					●				

● – required for diagnosis; ○ – may or may not be required

*References to the studies are listed in Table S1 in the **Online Supplementary Document**.

**Figure:**
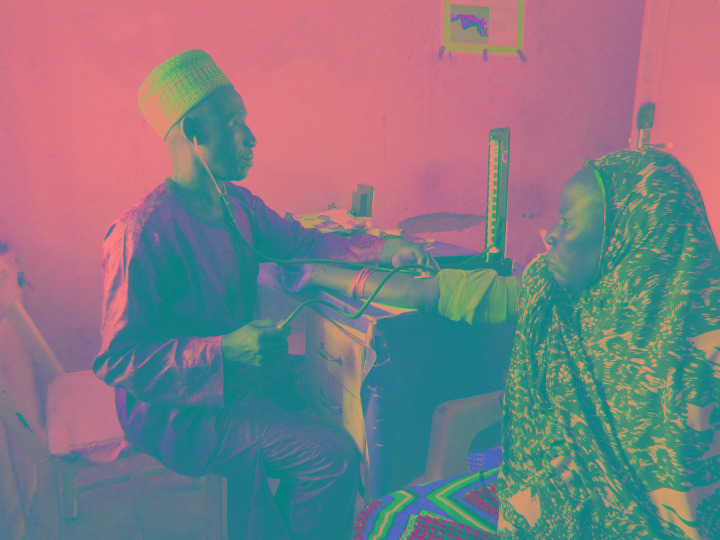
Photo: Healthcare provider measuring a pregnant woman’s blood pressure in Nigeria (from the collection of Karen Kirk, used with permission).

These terms include: essential hypertension, hypertension, hypertensive disorders of pregnancy, gestational hypertension, pregnancy-induced hypertension, gestational proteinuric hypertension, toxemia of pregnancy, pre-eclamptic toxemia, pre-eclampsia superimposed on essential hypertension, mild pre-eclampsia, pre-eclampsia, severe pre-eclampsia, imminent eclampsia, eclampsia, late postpartum eclampsia, hypertensive emergency, and Hemolysis, Elevated Liver enzymes, Low Platelet count (HELLP) Syndrome. The number of different definitions and diagnoses in these published articles fluctuates over time, with the greatest variety in the last five to seven years.

For an illustrative example, 25 articles (Nigeria = 10, Bangladesh = 15) set clinical requirements for diagnosis of “pre-eclampsia” in pregnant women. Thirteen definitions for PE (Nigeria = 6, Bangladesh = 7) require blood pressure (BP) measurements of over 140mmHg systolic blood pressure (sBP) and 90mmHg dBP as the diagnostic threshold for PE, while another four include an alternative, relative increase in sBP of >30mmHg and relative increase in dBP of >15mmHg; one of those definitions also diagnoses PE if dBP is 110mmHg at any time or if mean arterial pressure is >105mmHg. One definition uses 130mmHg as the threshold for sBP, and another sets its own standards using a range of BP levels (169 + 26.0 / 102 + 11.0mmHg) for diagnosing PE. Other definitions state broadly that PE is diagnosed when a patient has high BP, elevated BP, hypertension, and one describes PE as a “non-convulsive hypertensive disorder of pregnancy.”

The various clinical requirements and measurement methods for determining “proteinuria” further illustrate this variability. Proteinuria, typically included in diagnostic criteria for PE, is the occurrence of increased protein levels in urine that can be measured several different ways: via dipstick test (requiring either 2+ or 3+ results) or through laboratory analysis of 24-hour, 12-hour, or random samples. Common results for diagnosing proteinuria are >30 mg/dl in a random sample and >0.3g/L in 24-hour sample. Only one of the 25 definitions for PE did not mention proteinuria.

The literature from these countries exhibits further inconsistency on timing of diagnosis. Most definitions require diagnosis in the second half of pregnancy (at least 20 weeks), one requires 22 weeks, and another mandates the third trimester (27 weeks). Fourteen definitions do not specify when PE can be diagnosed, which could be due to the notion that “pre-eclampsia” implies diagnosis after 20 weeks; this omission does not necessary reflect lack of knowledge, since 20 weeks gestation is the generally-accepted global standard for PE diagnosis.

The differentiation among eclampsia, mild PE, and severe PE shows similar significant variations in definitions. Diagnosis criteria for mild PE and severe PE can include, or not: dizziness, headache, edema (swelling) of face, hands, and legs, epigastric (upper abdominal) pain, visual disturbances, pulmonary edema (excess fluid in lungs), hyperreflexia (overactive reflex), oliguria (low urine output), reduction in plasma volume, liver and renal function abnormalities, organ dysfunction, and coma. Definitions of “mild pre-eclampsia” demonstrate similar required BP levels, although not identical. From Bangladesh, one requires only dBP>90mmHg, and three use the combined >140/90mmHg to diagnose mild PE. From Nigeria, definitions for mild PE are more complicated. Osinubi et al. use >140/90mmHg or dBP 110 > 90mmHg and Okafor et al. use 160/110 > 140/90mmHg – or an increase in 30mmHg sBP or 15mmHg dBP (Table S1 in the **Online Supplementary Document**).

All six papers defining “mild pre-eclampsia” also define “severe pre-eclampsia;” more than fifty papers in the review provide definitions for “severe pre-eclampsia.” There is more consistency for BP levels among clinical definitions of “severe pre-eclampsia.” More than half require >160/110mmHg for diagnosis of severe PE, three only mention requiring dBP>110mmHg, one requires either >160mmHg sBP or >110mmHg dBP, while only one defines “severe pre-eclampsia” as requiring >90mmHg dBP.

The remaining terms appear with less frequency, perhaps demonstrating an evolution in how specific terms are used over time. “Toxemia” and its variations, including “pre-eclamptic toxemia” and “toxemia of pregnancy,” still appear in published literature, although they are most frequently equated with “pre-eclampsia.” Pregnancy-induced hypertension – another term not frequently defined – appears in the literature, but is equated with other terms including gestational hypertension, PE, severe PE, and eclampsia.

## CHALLENGE

These inconsistent and contradictory inclusion and exclusion criteria and differences in signs and symptoms for each classification impede timely prevention, detection, and management of HDPs. The variety of existing terms, including descriptors such as “mild,” “severe,” and “superimposed,” implies significant distinctions in treatment, and given the importance of screening, diagnosing, and managing these conditions early, these terms are too specific. A woman diagnosed with mild PE should receive the same attention, monitoring, and clinical tests as a woman diagnosed with PE or severe PE – either woman’s condition could worsen suddenly. The International Society for the Study of Hypertension in Pregnancy expressed this sentiment in a 2013 statement paper [5], but despite this, “mild” and “severe” distinctions are still in use. The lack of understanding of these conditions’ causation is a core challenge in effectively preventing, detecting, and diagnosing HDPs. Further research is consequential to improve health outcomes for women with HDPs and their babies.

The overabundance of terms and signs and symptoms associated with these conditions elicits confusion and low confidence among health workers for accurately diagnosing and managing these life-threating conditions. Lower cadre providers, especially those working in low- and middle-income countries (LMICs) like Bangladesh and Nigeria, who less frequently see cases of HDP may struggle to recall the various terms, corresponding diagnoses, and treatment protocols. Results from landscape analyses on PE/E in Nigeria and Bangladesh found that providers were more successful at diagnosing PE (Nigeria: 64%) or gestational hypertension (Bangladesh: 80%) and eclampsia (Nigeria: 81%, Bangladesh: 97%), but their abilities of defining severe PE (The assessment defined severe PE as being associated with “severe headache, changes in vision (including temporary loss of vision/blurred vision, light sensitivity, seeing spots), upper abdominal pain (usually under rips on right side, nausea/vomiting, dizziness), and decreased urine output”) were much lower (Nigeria: 9%, Bangladesh: 56%). This gap in provider knowledge suggests a lack of understanding of distinctions between the many terms currently used for HDPs.

## MAKING IMPROVEMENTS

The confusion around classifications and diagnostic requirements for HDPs is widespread beyond the literature from Bangladesh and Nigeria. Definitions and clinical guidelines are a topic of much debate, even among experts. Gillon et al.’s systematic review of international clinical practice guidelines for HDPs identified inconsistencies among definitions for PE and severe PE, “non-severe hypertension” BP, timing of delivery for women with PE and severe PE, magnesium sulfate administration in non-severe pre-eclamptic patients, and postpartum maternal monitoring [6]. Inconsistencies in Bazzano’s assessment include differences in weeks of high BP continuation after delivery for gestational or chronic hypertension determination, timing of corticosteroids for pre-term PE, and beginning high BP treatment with antihypertensive drugs [7].

Some efforts to provide clarity for defining and diagnosing HDPs use various biomarkers in tandem with BP levels [8] or define sub-types of PE based upon placental growth factor concentrations [9]. Recently, scientists studying these conditions’ causes have been debating differences in etiology of early-onset and late-onset PE. Alici Davutoğlu et al. identified a biomarker, serum progranulin, in significantly higher levels among women with late-onset than with early-onset PE [10]. In another study, Laskowska – who defines “early-onset” PE as prior to 34 weeks – identified an enzyme, MMP-3, as present only in women with early-onset PE [11]. In a review on these issues, Magee et al. identifies “areas of consistency” for hypertensive disorder types and diagnostic criteria during pregnancy, with remaining inconsistencies requiring further evaluation [12].

## CONCLUSION

Definitions for HDPs were found to be inconsistent and sometimes contradictory in two disparate countries like Bangladesh and Nigeria; it is reasonable to conclude that these challenges exist elsewhere. In LMICs, sub-classifications within HDPs have epidemiologic rather than diagnostic value. For researchers, collecting detailed data of clinical expressions of HDPs could perhaps illuminate hidden causes. In practice, however, overly-specific signs and symptoms distract, rather than clarify, diagnosis for women in potentially dire circumstances.

Moving beyond the implication that interventions vary significantly for each HDP’s classification has the potential to increase health care providers’ abilities to identify women at risk of developing these life-threatening conditions. Fewer and simpler classifications can enhance provider understanding (at any level of the health system) and improve diagnosis and case monitoring, provision of essential care, referral, and community knowledge to recognize the signs and symptoms of PE/E.

To save women’s lives, we must work together, globally, to create and agree upon specific, clear, and universal clinical definitions that ensure primary care providers worldwide can appropriately diagnose and manage the symptoms of PE/E.

## Additional material

Online Supplementary Document
