# Supplementary material for: “What’s in a name?”: Exploring inconsistent and contradictory definitions and clinical guidelines for hypertensive disorders of pregnancy from published literature from Nigeria and Bangladesh
Source: J Glob Health. 2020 Mar 14;10(1):010306. doi: 10.7189/jogh.10.010306 (PMC7100861; doi:10.7189/jogh.10.010306)
Supplement: Online Supplementary Document [file jogh-10-010306-s001.pdf]

SUPPLEMENTAL REFERENCES TABLE

Akiibinu MO, Kolawole TO, Ekun OA, Akiibinu SO. Metabolic dysfunctions in Nigerian pre-eclamptics. Arch Gynecol Obstet. 2013 Nov;288(5):1021–6.
Akter S, Sultana S, Dabee SR. Association of Hyperuricaemia with Perinatal Outcome in Pregnancy Induced Hypertension. J Bangladesh Coll Physicians Surg. 2014;32(3).
Arinola G, Arowojolu A, Bamgboye A, Akinwale A, Adeniyi A. Serum concentrations of immunoglobulins and acute phase proteins in Nigerian women with preeclampsia. Reprod Biol. 2006 Nov;6(3):265–74.
Begum MR, Quadir E, Begum A, Akhter S, Rahman K. Management of hypertensive emergencies of pregnancy by hydralazine bolus injection vs continuous drip - A comparative study. MEDSCAPE WOMENS Heal. 2002;7(5):6–11.
Begum N, Ferdousi R. Study of Gross Anatomy of Human Placenta in Pregnancy Induced Hypertension. JAFMC Bangladesh. 2014;10(2):55–61.
Begum S, Islam F, Jahan AA. Feto-maternal Outcomes in Cesarean Section Compared to Vaginal Delivery in Eclamptic Patients in a Tertiary Level Hospital. J Enam Med Coll. 2013;3(2):77–83.
Begum Z, Ara I, Tanira S, Keya K. the Association Between Serum Beta- Human Chorionic Gonadotropin and Preeclampsia. J Dhaka Med Coll. 2014;23(1):89–93.
Begum Z, Ara I, Tanira S, Keya K. THE ASSOCIATION BETWEEN SERUM BETA- HUMAN CHORIONIC GONADOTROPIN AND PREECLAMPSIA. J Dhaka Med Coll. 2014;23(1):89–93.
Chigbu CO, Okezie OA, Odugu BU. Women in southern Nigeria with change in paternity do not have increased incidence of pre-eclampsia. J Obstet Gynaecol. 2009 Feb;29(2):94–7.
Ebeigbe P, Aziken M. Early onset pregnancy induced hypertension/eclampsia in Benin City, Nigeria. Niger J Clin Pract [Internet]. 2010 [cited 2015 Apr 30]; Available from: http://www.ajol.info/index.php/njcp/article/view/64136
Fatima N, Islam F, Noor L, Das SR, Zeba D, Zesmin F. Serum Ferritin in Preeclampsia and Eclampsia : A Case Control Study. Faridpur Med Coll J. 2013;8(1):18–21.
Ferdausi M, Khatun M, Ma Y, Rahman A, Rahman Z. Association between High Serum Homocystine and Preeclampsia. J Shaheed Suhrawardy Med Coll. 2013;5(1):21–5.
Fronczak N, Antelman G, Moran AC, Caulfield LE, Baqui AH. Delivery-related complications and early postpartum morbidity in Dhaka, Bangladesh. Int J Gynaecol Obstet [Internet]. 2005 Dec [cited 2015 Jun 4];91(3):271–8. Available from: http://www.sciencedirect.com/science/article/pii/S0020729205004972
Guerrier G, Oluyide B, Keramarou M, Grais RF. Factors associated with severe preeclampsia and eclampsia in Jahun, Nigeria. Int J Womens Health. 2013;5(1):509–13.

Habib SAJ, Sultana S. a Study of Late Postpartum Eclampsia- 178 Cases. Bangladesh Med J. 2008;37(2):52–5.

Haque MM, Moghal MMR, Sarwar MS, Anonna SN, Akter M, Karmakar P, et al. Low serum selenium concentration is associated with preeclampsia in pregnant women from Bangladesh. J Trace Elem Med Biol [Internet]. 2016 Jan;33:21–5. Available from: http://www.sciencedirect.com/science/article/pii/S0946672X15300262

Hassan M, Begum M, Haque SMZ, Jahan N, Yasmeen BHN, Mannan A, et al. Immediate Outcome of Neonates with Maternal Hypertensive Disorder of Pregnancy at a Neonatal Intensive Care Unit. North Int Med Coll J. 2015;6(2):57–60.

Herrera JA, Arévalo-Herrera M, Shahabuddin AKM, Ersheng G, Herrera S, Garcia RG, et al. Calcium and conjugated linoleic acid reduces pregnancy-induced hypertension and decreases intracellular calcium in lymphocytes. Am J Hypertens [Internet]. 2006 Apr [cited 2015 Jun 4];19(4):381–7. Available from: http://www.sciencedirect.com/science/article/pii/S0895706105013105

Herrera JA, Shahabuddin AKM, Ersheng G, Wei Y, Garcia RG, López-Jaramillo P. Calcium plus linoleic acid therapy for pregnancy-induced hypertension. Int J Gynaecol Obstet [Internet]. 2005 Dec [cited 2015 Jun 4];91(3):221–7. Available from: http://www.sciencedirect.com/science/article/pii/S0020729205004844

Huda FA, Ahmed A, Dasgupta SK, Jahan M, Ferdous J, Koblinsky M, et al. Profile of maternal and foetal complications during labour and delivery among women giving birth in hospitals in Matlab and Chandpur, Bangladesh. J Heal Popul Nutr. 2012;30(2):131–42.

Ikechukwu IC, Ojareva OIA, Ibhagbemien AJ, Okhoaretor OF, Oluwatomi OB, Akhalufo OS, et al. Blood lead, calcium, and phosphorus in women with preeclampsia in Edo State, Nigeria. Arch Environ Occup Health. 2012;67(3):163–9.

Jafrin W, Mia AR, Chakraborty PK, Hoque MR, Paul UK, Shaha KR, et al. An evaluation of serum magnesium status in pre-eclampsia compared to the normal pregnancy. Mymensingh Med J. 2014 Oct;23(4):649–53.

Jahan K, Parvin M, Mafiz AI. Studies on Haemoglobin , Serum Calcium and Ascorbic Acid Level in Normal Pregnant , Pre-Eclamptic and Eclamptic Patients. Bangladesh J Nutr. 2011;24–25:7–14.

Jesmin S, Jahan S, Khan M, Sultana N, Jerin J, Habib S, et al. The Incidence , Predisposing Factors , Complications and Outcome of Preeclampsia in Diabetic Pregnancy. Bioderm Med J. 2011;1(1):10–4.

Khatun A, Latifa S, Shahla K, Anowara B, Suhrab A, Rashid HU, et al. Assessment of renal insufficiency in gestational proteinuric hypertension in third trimester pregnancy. Bangladesh Med Res Counc Bull. 2003 Dec;29(3):103–12.

Khatun S, Kanayama N, Belayet HM, Bhuiyan AB, Jahan S, Begum A, et al. Increased concentrations of plasma neuropeptide Y in patients with eclampsia and preeclampsia☆☆☆. Am J Obstet Gynecol [Internet]. 2000;182(4):896–900. Available from:

http://www.sciencedirect.com/science/article/pii/S0002937800703425
Kishwara S, Tanira S, Omar E, Wazed F, Ara S. Effects of Preeclampsia on Perinatal Outcome - A study done in the specialized urban hospital set up in Bangladesh. Bangladesh J Med Sci. 2011;40(1):33–6.
Makinde ON, Adegoke OA, Adediran IA, Ndububa DA, Adeyemi AB, Owolabi AT, et al. HELLP syndrome: the experience at Ile-Ife, Nigeria. J Obstet Gynaecol. 2009 Apr;29(3):195–9.
Makinde ON. The Contribution of Severe Pre-Eclampsia and Eclampsia to Perinatal Mortality in a Nigerian Teaching Hospital. In: Ezechi DO, editor. Perinatal Mortality. 2011.
Nessa SST, Polash MMI, Islam M, Anam AM, Bhuiyan MMR. HELLP syndrome : An experience of treating with plasma exchange. Bangladesh Crit Care J. 2013;1(2):102–3.
Okafor U, Efetie E. Anaesthetic management of patients with pre-eclampsia/eclampsia and perinatal outcome. J Matern Neonatal Med [Internet]. 2009 [cited 2015 Apr 30];22(8):688–92. Available from: http://informahealthcare.com/doi/abs/10.1080/14767050902994473
Okafor U. Maternal and perinatal outcome after caesarean delivery in preeclampsia or eclampsia in Enugu, Nigeria: four years on. Int J Obstet Anesth [Internet]. 2009 [cited 2015 Apr 30].
Okusanya BO, Garba KD, Ibrahim HM. The efficacy of intramuscular loading dose of MgSO4 in severe pre-eclampsia/ eclampsia at a tertiary referral centre in Northwest Nigeria. Niger Postgrad Med J. 2012 Jun;19(2):77–82.
Okusanya BO, Garba KK, Ibrahim HM. The efficacy of 10gram intramuscular loading dose of MgSO(4) in severe preeclampsia/ eclampsia at a tertiary referral centre in Northwest Nigeria. Niger Postgrad Med J. 2012 Sep;19(3):143–8.
Olusanya BO, Solanke OA. Perinatal outcomes associated with maternal hypertensive disorders of pregnancy in a developing country. Hypertens pregnancy. 2012;31(1):120–30.
Onah H. Prognostic value of absolute versus relative rise of blood pressure in pregnancy. Afr J Reprod Health. 2002 Apr;6(1):32–40.
Onyiriuka AN, Okolo AA. Neonatal morbidity pattern in infants born in Benin City to Nigerian mothers with hypertensive disorders in pregnancy. Niger J Clin Pract. 2007 Dec;10(4):294–9.
Osinubi A, Ajayi G, Adegbola O. Effect of normal and pre-eclamptic pregnancies on plasma cholinesterase in Nigerian women. African J Endocrinol Metab. 2009;8(1):1–3.
Rahman S, Gupta R Das, Islam N, Das A, Shaha AK, Khan MAI, et al. Pregnancy related acute renal failure in a tertiary care hospital in Bangladesh. J Med [Internet]. 2012;13(2):129–32. Available from: http://www.banglajol.info/index.php/JOM/article/view/12739/9183
Razzaque A, Da Vanzo J, Rahman M, Gausia K, Hale L, Khan MA, et al. Pregnancy spacing

and maternal morbidity in Matlab, Bangladesh. Int J Gynaecol Obstet [Internet]. 2005 Apr [cited 2015 Jun 4];89 Suppl 1:S41-9. Available from: http://www.sciencedirect.com/science/article/pii/S0020729205000408
Salako BL, Olayemi O, Odukogbe A-TA, Adedapo KS, Aimakhu CO, Alu FE, et al. Microalbuminuria in pregnancy as a predictor of preeclampsia and eclampsia. West Afr J Med. 2003 Dec;22(4):295–300.
Sayeed MA, Mahtab H, Khanam PA, Begum R, Banu A, Azad Khan AK. Diabetes and hypertension in pregnancy in a rural community of Bangladesh: a population-based study. Diabet Med. 2005 Sep;22(9):1267–71.
Sufrin S, Nessa A, Islam M, Khatun A, Husain M, Khatun N, et al. Blood Pressure in Third Trimester of Pregnancy. Mymensingh Med J. 2016;25(1):18–22.
von Dadelszen P, Ayres de Campos D, Barivalala W. Classification of the hypertensive disorders of pregnancy. In: The FIGO Textbook of Pregnancy Hypertension. London; 2016. p. 33–61.
Yoshimura T, Chowdhury FA, Yoshimura M, Okamura H. Genetic and environmental contributions to severe preeclampsia: lack of association with the endothelial nitric oxide synthase Glu298Asp variant in a developing country. Gynecol Obstet Invest. 2003;56(1):10–3.
